# Internalized stigma among patients with schizophrenia in Ethiopia: a cross-sectional facility-based study

**DOI:** 10.1186/1471-244X-12-239

**Published:** 2012-12-29

**Authors:** Dereje Assefa, Teshome Shibre, Laura Asher, Abebaw Fekadu

**Affiliations:** 1 Amanuel Specialized Mental Hospital, Addis Ababa, Ethiopia; 2 Department of Psychiatry, School of Medicine, College of Health Sciences, Addis Ababa University, Addis Ababa, Ethiopia; 3 Department of Epidemiology and Public Health, University College London, London, UK; 4 King’s College London, Institute of Psychiatry, Department of Psychological Medicine, London, UK

**Keywords:** Internalized stigma, Schizophrenia, Adherence, Recovery, Ethiopia

## Abstract

**Background:**

Despite the potential impact on treatment adherence and recovery, there is a dearth of data on the extent and correlates of internalized stigma in patients with schizophrenia in low income countries. We conducted a study to determine the extent, domains and correlates of internalized stigma amongst outpatients with schizophrenia in Ethiopia.

**Methods:**

The study was a cross-sectional facility-based survey conducted at a specialist psychiatric hospital in Addis Ababa, Ethiopia. Consecutive consenting individuals with a diagnosis of schizophrenia were recruited and assessed using an Amharic version of the Internalized Stigma of Mental Illness (ISMI) scale.

**Results:**

Data were collected from 212 individuals, who were mostly single (71.2%), unemployed (70.3%) and male (65.1%). Nearly all participants (97.4%) expressed agreement to at least one stigma item contained in the ISMI; 46.7% had a moderate to high mean stigma score. Rural residence (OR = 5.67; 95% CI = 2.30, 13.00; p < 0.001), single marital status (OR = 3.39; 95% CI = 1.40, 8.22; p = 0.019) and having prominent psychotic symptoms (OR = 2.33; 95% CI = 1.17, 4.61; p = 0.016) were associated independently with a higher stigma score. Almost half of those who discontinued their treatment reported that they had done so because of perceived stigma. Those who had attempted suicide (45.3%) were more likely to have a high stigma score (OR = 2.29; 95% CI = 1.27, 4.11; p = 0.006). Over 60% of the variation in the experience of stigma was explained by four factors: social withdrawal (16.7%), perceived discrimination (14.1%), alienation (13.9%) and stereotype endorsement (12.7%).

**Conclusion:**

Internalized stigma is a major problem among persons with schizophrenia in this outpatient setting in Ethiopia. Internalized stigma has the potential to substantially affect adherence to medication and is likely to affect the recovery process.

## Background

Whilst most mental illnesses may be associated with some degree of stigma, schizophrenia has been described as “a modern day equivalent of leprosy”, conferring unparalleled social ostracism to the person with schizophrenia and their family [1]. Stigma comprises of three elements: problems of knowledge (misinformation), problems of attitudes (prejudice), and problems of behaviour (discrimination) [2].

Furthermore, two major classes of stigma are recognized: public stigma and personal stigma. Public stigma (or social stigma) typically describes the endorsement of stereotypes and enacted behaviours against people with mental illness by professionals and the general population [2,3]. Personal experiences of stigma, measured in those with mental illness, can be considered in three ways [3]:

(i) Perceived stigma: what the individual thinks are society’s beliefs about the stigmatized group

(ii) Experienced stigma: actual discrimination experienced

(iii) Self-stigma: a product of the internalization of public stigma

In internalized stigma or self-stigma, a gradual process of psychological assimilation of the public stereotypes towards mental illness is said to occur such that the person with mental illness progressively loses what they think they are and wish to be in the future [4]. In essence, the person not only believes that others think s/he is not worthwhile or should not, for example be married, but also believes that s/he is not actually worth much and should not be married. This changes fundamentally the perception the person has about themselves and leads to a change in the person’s behaviour in a way that matches the internalized perceptions. Until recently most research has focused on public stigma rather than internalized stigma [2]. Several studies have emphasized the negative consequences of such negative perceptions and behaviour, including loss of self-esteem and self-efficacy, disempowerment, demoralization and loss of income [5-8]. Clinically, higher depressive symptoms, increased suicidality and poorer medication adherence are also reported [9]. Because of the above consequences and related negative impacts such as social withdrawal, poor vocational functioning and worse quality of life [5-7,9-14], internalized stigma is emerging as a key factor that can hold back the recovery process.

Stigma is a major problem across different societies, but the particular manifestations of stigma may vary. Variation in the interaction between stigma and mental illness is evident in different settings [15]. Stigma is also considered an important explanatory factor for the better outcome of schizophrenia in low income settings [16]. Families in developing societies have been described as “supportive and tolerant” and that there is “little risk of prolonged rejection, isolation, segregation and institutionalisation” [17]. In the developed societies that emphasize individuality, it is proposed that loss of employment would mean loss of status and self-esteem and rejection. The person with the illness takes responsibility for the illness and its consequences. Although these principles are relevant, making broad generalizations cannot always hold true. For example, in cultures where stigma is considered to be a moral issue, it may threaten what “matters most” for those living in the local world [18,19]. Moreover, if the proposition about persons from low income setting taking less individual responsibility is correct, this is likely to be replaced by the responsibility they feel for their family. Thus a person from low income settings would feel responsible for the consequences of the illness on the family experiencing the illness not only as a personal tragedy but that they have caused disgrace to the family by becoming ill, and that the condition may have devastating consequences on the social standing of the family. Although our study has not explored these broader cultural issues, we consider that the level of stigma is likely to reflect the broader personal and family implications of having a mental illness.

Most of our understanding about internalized stigma originates from high-income countries. Yet the few available studies have demonstrated high levels of internalized stigma amongst patients with schizophrenia in some African countries: Ghana [20], Nigeria [21] and South Africa [22]. There has been no previous assessment of internalized stigma in Ethiopia. The only reported study concerning stigma in severe mental disorders in Ethiopia looked at perceived stigma among 178 carers of persons with schizophrenia and major affective disorders [23]. This study was part of a large community-based project in Southern Ethiopia [24]. About 75% of the caregivers reported experiencing stigma. Urban residence and older age were the main factors associated with higher experience of stigma. The finding suggests that the experience of internalized stigma may also be high [23].

Systematic investigation of the extent and correlates of internalized stigma is an essential part of planning for recovery programs for schizophrenia. This research is timely for the Ethiopian setting and other low-income country settings as mental health services are undergoing a shift towards primary health care and community settings [25]. Community-based rehabilitation (CBR) for people with schizophrenia, including social and vocational rehabilitation, will form part of the services under development. An understanding of the stigma and discrimination experienced by this group is vital to developing appropriate training materials for the community health workers who will deliver the CBR. Anti-stigma campaigns are gaining prominence in high-income countries [26]. For low-income countries to follow suit a more detailed understanding of the extent and nature of the stigma related to mental illness is required. This will also allow evaluation of the effectiveness of anti-stigma campaigns.

### Objectives

The primary objective of the study was to assess the extent of internalized stigma among patients with schizophrenia attending the outpatient department of a psychiatric hospital in Ethiopia. Related to this we aimed to determine which socio-demographic and clinical factors are associated with the experience of stigma and to explore the potential impact of stigma on medication adherence and risk behaviour; important factors that may affect the recovery process.

## Methods

### Study design and setting

The study was a facility-based cross-sectional survey conducted at a psychiatric hospital located in the capital city, Addis Ababa. The hospital is the oldest and largest public psychiatric hospital in Ethiopia. Patients from all over the country come to the hospital to receive treatment for severe mental disorders. The hospital service is organized under specialist programs or specialist case teams. Each case team is expected to have expert knowledge about the disorders it treats. Patients with schizophrenia are treated by psychoses case teams and this case team manages all individuals with schizophrenia referred or seen by the hospital. The hospital has an inpatient service with 280 bed and a large out-patient service, with around 115,000 outpatients visiting the hospital each year.

### Participants

Participants of this study were patients with a clinical diagnosis of schizophrenia, aged 18 years and above. Potential participants were selected by reviewing the medical records of patients attending regular follow-up appointments. Individuals with clinically established impairment of insight, a significant level of cognitive impairment or significant level of substance abuse in the previous three months were excluded based on a review of clinical records and clinical assessment by senior psychiatric residents. We focused on schizophrenia in order to have a relatively uniform group of participants. Individuals with substance abuse were excluded because of the potential impact of substance abuse on the experience of stigma.

### Assessment

The Internalized Stigma of Mental Illness (ISMI) Scale for the assessment of stigma [27] was translated into Amharic, the official language of Ethiopia. The ISMI scale is a widely used instrument consisting of 29 items, which have been grouped into five domains: alienation, stereotype endorsement, social withdrawal, perceived discrimination and stigma-resistance. The 29-item ISMI, and four of the five domains, have good levels of internal consistency and test-retest reliability [27]. However, the fifth subscale (stigma-resistance subscale) is conceptually different from the other parts of the scale and has lower internal consistency [28,29]. This subscale was therefore not used in our study. The 24-item ISMI is reported to have excellent internal consistency with an alpha of 0.91 and a test–retest correlation of 0.73 [27]. Each ISMI item contains a declarative statement about a potential stigma issue and participants respond to each statement by indicating their level of agreement: 1 = strongly disagree; 2 = disagree; 3 = agree; 4 = strongly agree.

In addition to the ISMI, participants were asked simple questions in relation to whether they experienced stigma within their family, in the neighbourhood and whether they felt disrespected by the mental health staff because of their illness.

Furthermore, we asked two questions on medication adherence and one on suicide attempts. The medication adherence question asked about history of non-adherence with psychotropic medications and whether the non-adherence behaviour was linked to stigma. Specifically we asked “Have you ever discontinued medication”? and “Did your experience of stigma contribute to your decision to discontinuing medication?”. The question on suicide simply asked whether the participant had felt so desperate that they had attempted suicide (“Have you ever felt so desperate that you even attempted to harm yourself or end your life?”). This question is similar to the single question used in asking about suicide attempt in the widely used structured diagnostic interview instrument of the World Health Organization, the Composite International Diagnostic Interview [30]. The occurrence of positive and negative symptoms was assessed using a checklist from the Diagnostic and Statistical Manual of Mental Disorders, Fourth edition (DSM-IV) [31] while the presence of extrapyramidal symptoms was assessed clinically.

### Instrument adaptation procedure

The 24-item ISMI scale, which has been validated and used in an outpatient setting for patients with schizophrenia [27], was translated and used in our study. After producing three independent Amharic versions by three senior psychiatry residents and a senior psychiatrist, a consensus version was developed in a group discussion involving two other senior psychiatrists. The consensus version was back-translated by a consultant psychiatrist. This was compared with the original consensus version, which required only minor modifications. Inter-rater reliability assessment was completed by the four psychiatry residents who participated in the administration of the instrument. The inter-rater agreement was good (Kappa = 0.76). The Cronbach’s alpha based on standardised items was also good (0.92). Before the study a pilot exercise was undertaken and necessary modifications were made.

### Administration of instruments

After inclusion criteria were met, all instruments were administered in the local language (*Amharic*) by senior psychiatric residents. Although the ISMI is self-administered instrument, the ISMI questions were directly asked by the residents. Because of the variable level of literacy it was deemed necessary to use interviewers who read the questions to the participants.

### Data analysis

Analyses were performed using SPSS version 17 and Stata version 11. Most predictive variables were categorized a priori. Age was also categorized into ten-year intervals before analysis was run in order to determine which age group, if any, would be more affected by the experience of internalized stigma. Mean scores for each ISMI item and domains, as well as the overall ISMI, were computed following the standard analytic methods for internalized stigma used in previous studies [32]. We extracted data from a European study [32] as a comparison for our findings. A binary logistic regression model was used to determine the association of selected socio-demographic and clinical factors with higher internalized stigma experience using the mean internalized stigma score as a cut off. Furthermore, for each ISMI item we calculated the proportion reporting ‘Any stigma’ (a response of “agree” or “strongly agree” on any stigma item) and ‘Strong stigma’ (a response of “strongly agree” only). Finally, exploratory factor analysis was completed using principal component analysis and varimax rotation to determine how the variability in the experience of internalized stigma might be explained. This method was preferred to a confirmatory factor analysis method because of the lack of previous studies in Ethiopia using the ISMI.

### Ethical considerations

The study proposal was initially approved by the scientific committee of the Department of Psychiatry, School of Medicine, College of Health Sciences, Addis Ababa University. Amanuel Hospital Ethics Committee granted further ethical approval. Informed consent was sought from each participant.

## Results

### Socio-demographic and clinical characteristics

There were 212 participants, the majority of whom were males (65.1%) and from urban areas (79.7%). The mean age of the participants was 33.3 years (SD 8.9). Nearly three-quarters (70.3%) were unemployed although most had some level of education, and 20.8% were college graduates. Only 25.9% were married (Table 1), with over half dependent on their family. Most of the participants had indications of severe illness (Table 2): 45.3% had attempted suicide at least once since the onset of their illness; 67.5% had a history of admission; and 55.7% had noticeable extra-pyramidal side effects. During the assessment 32.1% of the participants had uncontrolled psychotic symptoms; with either prominent positive or negative symptoms or a combination of these. Nearly two-thirds (60.4%) of participants admitted discontinuing their treatment. Sixty two percent of those who discontinued treatment attributed this to internalized stigma related to their illness.

**Table 1:** **Socio**-**demographic characteristics of participants**

**Variable**		**Total**	
		**N**	**%**
**Total**	– – – – – –	212	100
**Sex**	Male	138	65.1
	Female	74	34.9
**Age** (**years**)	<25	30	14.2
	25-34	89	42.0
	35-44	70	33.0
	≥45	23	10.8
**Residence**	Urban	169	79.7
	Rural	43	20.3
**Employment**	Employed	63	29.7
	Unemployed	149	70.3
**Ethnicity**	Amhara	85	40.1
	Oromo	50	23.6
	Gurage	52	24.5
	Others	25	11.8
**Educational level**	Non-literate	19	9.0
	Primary	30	14.2
	Secondary	119	56.1
	College	44	20.8
**Marital status**	Single	151	71.2
	Married	55	25.9
	Divorced	6	2.8

**Table 2:** Clinical characteristics of participants

**Variable name**		**Total**	
		**N**	**%**
**Total**	– – – – – –	212	100
**Suicide attempt**	Yes	96	45.3
	No	116	54.7
**History of admission**	Yes	143	67.5
	No	69	32.5
**History of EPSE**	Yes	118	55.7
	No	94	44.3
**Medication non**-**adherence**	Yes	128	60.4
	No	84	39.6
**Contribution of stigma to non**-**adherence** (**N** = **128**)**	Yes	62	48.4
	No	66	51.6
**Prominent psychotic symptoms**	Yes	68	32.1
	No	144	67.9

Abbreviations: EPSE = Extrapyramidal side effects;

**Those without history of non-adherence (N = 84) excluded.

### ISMI Factor domains

Basic standards for conducting factor analysis were met. The Kaiser-Meyer-Olkin measure of sampling adequacy was 0.903, well above the recommended minimum of 0.6, and Bartlett’s test of sphericity was significant (χ^2^ (276) = 2496.0, *p* < 0.001). Finally, the communalities were all above 0.43. Only factors with Eigen values of one or above were included. Four factors were identified from the factor analysis: social withdrawal, perceived discrimination, alienation and stereotype endorsement. These factors explained 61.9% of the total variance: social withdrawal 16.7%, perceived discrimination 14.1%, alienation 37.9% and stereotype endorsement 12.4% (Table 3).

**Table 3:** **Factor loadings of the internalized stigma of mental illness scale based on principal components analysis and varimax rotation method** (**values under**.**3 suppressed**)

**Stigma items**	**Factors**			
	**Social withdrawal**	**Perceived discrimination**	**Alienation**	**Stereotyped endorsement**
**Out of place** (I feel out of place in the world because I have a mental illness)				.46
**Spoiled life** (Having a mental illness has spoiled my life)				.72
**Others can**’**t understand** (People without mental illness could not possibly understand me)		.36		.53
**Embarrassment** (I am embarrassed or ashamed that I have a mental illness)				.73
**Disappointment** (I am disappointed in myself for having a mental illness.)				.74
**Feel inferior** (I feel inferior to others who don’t have a mental illness)				.73
**Stereotype belief** (Stereotypes about the mentally ill apply to me)	.32		.50	
**Known by looks** (People can tell that I have a mental illness by the way I look)		.32	.47	
**Violence** (Mentally ill people tend to be violent)			.58	
**Decisions** (Because I have a mental illness, I need others to make most decisions for me)	.31		.57	
**Rewarding Life** (People with mental illness cannot live a good, rewarding life)			.68	
**Marriage** (Mentally ill people shouldn’t get married)			.74	
**Contribution** (I can’t contribute anything to society because I have a mental illness)			.67	
**Discrimination** (People discriminate against me because I have a mental illness)		.70		
**Achievement** (Others think that I can’t achieve much in life because I have a mental illness)		.78		
**Ignored** (People ignore me or take me less seriously just because I have a mental illness)		.78		
**Patronized** (People often patronize me, or treat me like a child, just because I have a mental illness)		.76		
**People disinterested** (Nobody would be interested in getting close to me because I have a mental illness)		.64		
**Burden others** (I don’t talk about myself much because I don’t want to burden others with my mental illness)	– – – –	– – –	– – –	– – –
**Reduced socialisation** (I don’t socialize as much as I used to because my mental illness might make me look or behave "weird")	.79	– – –		
**Isolated** (Negative stereotypes against people with mental illness like myself keep me isolated from the "normal" world)	.82			
**Protect family** (I stay away from social situations in order to protect my family or friends from embarrassment)	.71			
**Feel inadequate** (Being around people who don’t have a mental illness makes me feel out of place or inadequate)	.82			
**Rejection** (I avoid getting close to people who don't have a mental illness to avoid rejection )	.85			
Eigen values	8.6	2.0	1.8	1.5
Percentage of total variance	16.7	14.1	13.9	12.4
Number of test variables	5	5	7	6

### Prevalence of internalized stigma

**Figure 1:**
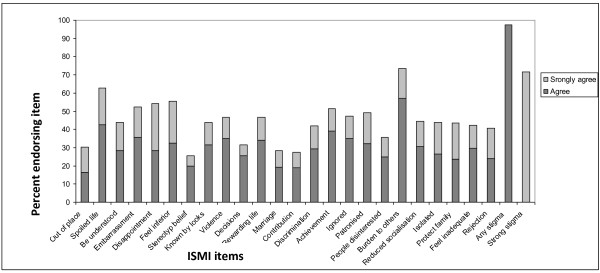
**Proportion of patients with schizophrenia reporting internalized stigma experience according to the degree of endorsement (agreement).** ISMI = Internalized Stigma of Mental Illness.

Overall 97.4% of participants reported experiencing at least one internalized stigma item at the time of the interview. Nearly three-quarters of respondents (71%) expressed strong agreement to at least one internalized stigma item (Figure 1). Some patients experienced stigma within the family (24.5%) and neighborhood (17%). Occasional disrespect from mental health staff was reported by 31.6% of the participants. The overall mean internalized stigma score across all four domains was between 2.3 and 2.5 and comparison with data from the above European study indicates clear similarity (Table 4) in the experience of internalized stigma. Based on the mean score, and using similar score categories to the European study (<2 minimal stigma, 2-2.5 low stigma, 2.5-3 moderate stigma, 3+ strong stigma) [32], 46.7% of participants had moderate or high stigma score.

**Table 4:** Comparison of mean scores of stigma domains between patients with schizophrenia in Ethiopia and Europe

**Stigma domain**	**Stigma score**			
	**Ethiopia**		Europe*	
	**Mean**	**SD**	**Mean**	**SD**
Social withdrawal	2.4	0.9	2.4	0.6
Discrimination	2.5	0.7	2.4	0.6
Alienation	2.3	0.6	2.5	0.7
Stereotyped endorsement	2.5	0.6	2.1	0.5

* European data extracted from Brohan et al 2010 [29].

### Association of sociodemographic and clinical factors with internalized stigma

The factors independently associated with higher internalized stigma score (moderate to high mean score) were rural residence (OR = 5.67; 95% CI = 2.30, 13.00; p < 0.001), single marital status (OR = 3.39; 95% CI = 1.40, 8.22; p = 0.019) and having prominent psychotic symptoms (OR = 2.69; 95% CI = 1.36, 5.33; p = 0.004) (Table 5). Although college education was associated negatively with internalized stigma, this was not statistically significant (p = 0.057). However, lower internalized stigma was associated with increasing levels of education (Test for trend: Z = -2.07; P = 0.04). Rural residence, single marital status and psychotic symptoms were also important predictors of the individual stigma domains. Education was significantly and negatively associated with the domain of “stereotype endorsement” only (OR = 0.16; 95% CI = 0.16, 0.83; p = 0.016).

**Table 5:** **Sociodemographic and clinical factors associated with moderate or high stigma scores** (**mean score of 2**.**5 and above**)

**Variables**		N*	**Unadjusted model**			**Fully adjusted model**			
			**OR**	**95%CI**		**OR**	**95%CI**		**P value**
Sex	Male	66/138	1.14	0.50	2.01	1.12	0.58	2.15	0.737
	Female	33/74	Ref.						
Marital status	Single**	79/157	1.77	0.94	3.34	3.39	1.40	8.22	0.019
	Married	20/55	Ref.						
Education	College graduate	18/44	075	0.37	1.52	0.44	0.19	1.02	0.057
	Non-literate	11/19	1.50	0.56	3.98	1.47	0.43	5.06	0.539
	Primary	13/30	0.83	0.37	1.86	1.16	0.45	2.98	0.756
	Secondary	57/119	Ref.						
Religion	Moslem	20/51	0.71	0.36	1.38	0.59	0.26	1.35	0.210
	Others	25/48	1.19	0.60	2.34	1.55	0.70	3.47	0.284
	Orthodox	54/113	Ref.						
Age (years)	<25	13/30	0.99	0.33	2.97	0.54	1.15	2.03	0.365
	25-34	43/89	1.22	0.48	3.06	0.77	0.17	1.69	0.633
	35-44	33/70	1.16	0.45	2.99	0.71	0.23	1.88	0.543
	≥45	10/23	Ref.						
Residence	Rural	31/43	3.84	1.84	8.00	5.67	2.30	13.00	<0.001
	Urban	68/169	Ref.						
Employment	Unemployed	79/149	1.04	0.56	1.88	0.66	0.33	1.35	0.232
	Employed	29/63	Ref.						
Ethnicity	Amhara	43/85	1.79	0.96	3.36	1.88	0.87	4.08	0.110
	Oromo	28/50	2.23	1.08	4.60	1.73	0.75	4.01	0.201
	Others	28/77	Ref.						
Prominent psychotic symptoms	Yes	68	2.17	1.19	3.93	2.69	1.36	5.33	0.004
	No	144	Ref.						

* = indicates number with moderate to high stigma/total in category.

** = includes 6 divorced individuals.

There was evidence of an association between a history of suicide attempt and high internalized stigma score which persisted after adjusting for sex, age, religion, residence, employment and adherence to medication (OR 2.29; 95% CI 1.27, 4.11; p = 0.006). The association between medication non-adherence and suicide attempt was of borderline statistical significance (OR 1.81; 95% CI 1.00, 3.27; p = 0.050). For those discontinuing their medication, stigma was the most important factor associated with discontinuation of their medication (OR = 5.03; 95% CI = 1.98, 12.78; p = 0.001) after controlling for the experience of extrapyramidal side effects, educational status, symptom level, residence, employment, age and sex.

## Discussion

### Prevalence of internalized stigma

This study demonstrates the high burden of internalized stigma among patients with schizophrenia attending a specialist hospital in Ethiopia. Almost all participants had experienced at least some level of internalized stigma and about three-quarters endorsed strongly at least one internalized stigma item. The experience of stigma in each stigma domain was also high (50 to 72%) with no significant difference within categories.

This is the first study in Ethiopia to investigate internalized stigma among patients with schizophrenia and the second addressing the issue of stigma among patients with schizophrenia. This study is also one of the very few studies dealing with internalized stigma from sub-Saharan Africa.

The current study replicates findings from previous studies of stigma relating to mental illness from sub-Saharan Africa [33-35] and Asia [36]. This study also demonstrates that, in Ethiopia, the level of internalized stigma experienced by patients with schizophrenia is comparable to that of patients from Europe [32] across all four internalized stigma domains (Table 4). From the perspective of the mean score categories, at least a comparable proportion of our patients have moderate to high stigma scores (46.7%) compared to patients from Europe (41.7%) [32]. However, it may be argued that participants in our study, coming predominantly from urban areas, with a literacy rate of over 90% and a high rate of treatment receipt (Table 1 and Table 2), are more typical of patients with schizophrenia from Europe than a low-income country. In Ethiopia only 16% of the population lives in cities and towns [37], just 36% are literate [38] and only about 10% of those with severe mental disorders have a lifetime history of receipt of treatment [39,40]. Nevertheless, in our study patients coming from rural areas were more likely to have higher stigma scores. Additionally, there was a trend for higher levels of education (or more years in school) to be associated with lower stigma score. Therefore, in this study population, having characteristics commonly found among patients from high-income settings (e.g., urban residence and higher educational achievement) may in fact confer a degree of protection against stigma. The major limitation of this comparison is that the European data is based on a much larger sample of participants (n = 1229) from 14 European countries.

One of the explanations for the apparently favourable outcome of schizophrenia in low-income countries, is the presumed low prevalence of stigma in these settings [41]. This assertion of improved outcome in low-income settings has long been disputed [42], yet even if it is accurate, the explanation for the better outcome in low income settings is unlikely to be related to low levels of stigma. There is now consistent evidence to suggest that the level of stigma in low income settings is at least as high as that found in high income settings. The nature of stigma, as shown in the ISMI sub-domains and factor analysis, also appears consistent with that reported in European studies [27-29].

### Internalized stigma and possible impact upon the recovery process

Findings from this study support the hypothesis that stigma is an important obstacle in the recovery process. Nearly half of all patients who were non-adherent to their medication attributed their decision to discontinue treatment to stigma. This group was predominantly among those with higher level of stigma score. Treatment adherence is the most important predictor of positive outcome in severe mental disorders in low-income countries [43,44]. Stigma may impact upon adherence through various psychological mechanisms such as loss of self-esteem and self-efficacy, demoralization, hopelessness and depression [4,28,45]. Stigma also leads to limited achievement of rehabilitation goals such as living independently [46-49]. The role of these factors is not assessed in this study but would be of importance to do so in this setting.

Additionally we found that a higher stigma score was associated robustly with a history of suicide attempt, an important negative outcome of severe mental disorders. Therefore, tackling stigma should be an important public mental health intervention priority in low income countries. Strategies to address stigma should be incorporated into initiatives to scale up mental health services in low and middle-income countries [50].

### Factors associated with internalized stigma

A higher level of internalized stigma was associated with three factors: having rural residence, single marital status and prominent psychotic symptoms. Rural residence has been found previously to be associated with higher stigma [51], and was attributed to lack of privacy and anonymity in small rural communities. Such lack of privacy and anonymity may have an impact on help seeking, for example, by families attempting to hide the patient away. This is particularly important in low income settings where families play a key role in patients’ receipt of treatment.

The association of internalized stigma with psychotic symptoms may be attributed to “reverse causality”. Patients with higher levels of psychotic symptoms may be more likely to perceive negative attitudes from others, related to their psychopathology. On the other hand, uncontrolled symptoms may attract more attention to the affected person and this may lead to discrimination.

There are two pathways in which uncontrolled symptoms may act as barriers to recovery: through reduced adherence to treatment because of denial and fear of stigma, and the psychological impact of stigma. Although not robustly associated with internalized stigma, the trend in this study is that education may be protective against stigma, particularly stereotyped endorsement. Again this is in line with findings of studies in other settings [52-54].

### Limitations

The study design has inherent weaknesses, which were difficult to avoid because of feasibility issues. The cross-sectional design meant that it was not possible to infer causality from the findings. The lack of longitudinal analysis in relation to factors associated with internalized stigma has been highlighted previously [55]. The study setting and sample were not completely representative of the patient population with schizophrenia. Only patients attending an out-patient service of a referral hospital participated in the study and those with history of substance abuse were excluded. Patients who were not fluent in Amharic were excluded due to lack of interpreters. Patients with significant cognitive impairment and impaired insight were also excluded. These exclusions do reduce the generalisability of the findings. However, comparison with a previous report describing the characteristics of patients admitted over a one year period in the same specialist hospital as our participants came from indicates general comparability with patients being admitted [56]. Finally, some of the outcomes were assessed with single questions, for example treatment adherence and suicide attempt. The question in relation to treatment adherence was close-ended question and framed in a way that could have led some patients to respond affirmatively.

## Conclusions

This study suggests that a significant burden of stigma is borne by patients with schizophrenia in Ethiopia. There is sufficient data now from low-income countries to suggest that a low prevalence of stigma cannot explain the presumed favourable outcome of schizophrenia in low-income countries. The current report, along with other similar data from Ethiopia and other low-income countries, supports the need to incorporate culturally appropriate methods of addressing internalized stigma into rehabilitation packages for this group. Particular attention should be paid to the potential for self-stigma to inhibit help seeking and treatment adherence.

## Competing interests

All the authors declared no competing interest.

## Authors’ contributions

DA, TS and AF were involved in the design of the study and all authors were involved in the analysis of data and preparation of the manuscript. All authors read and approved the final manuscript.

## Pre-publication history

The pre-publication history for this paper can be accessed here:

http://www.biomedcentral.com/1471-244X/12/239/prepub
